# Environmental determinant of malaria cases among travellers

**DOI:** 10.1186/1475-2875-12-87

**Published:** 2013-03-04

**Authors:** Gaëtan Texier, Vanessa Machault, Meili Barragti, Jean-Paul Boutin, Christophe Rogier

**Affiliations:** 1 Public Health and Epidemiology Centre of the French Army (CESPA) & SESSTIM UMR912, Allée du Médecin Colonel Jamot, Parc du Pharo, BP60109, 13262 Marseille cedex 07, France; 2 Centre Pasteur du Cameroun BP 1274, Yaoundé, Cameroun, France; 3 Institute for Biomedical Research of the French Army (IRBA) & URMITE UMR6236, Allée du Médecin Colonel Jamot, Parc du Pharo, BP60109, Marseille cedex 07, 13262, France; 4 Observatoire Midi-Pyrénées - Laboratoire d’Aérologie, Centre National de la Recherche Scientifique - Université Paul Sabatier, 14 avenue Edouard, Belin, Toulouse, 31400, France; 5 SUPAGRO, INRA, UMR729 MISTEA, Montpellier, F-34060, France; 6 Institut Pasteur de Madagascar, B.P. 1274, 101, Antananarivo, Madagascar

**Keywords:** Malaria, Environment, Remote sensing technology, Forecasting, Travel

## Abstract

**Background:**

Approximately 125 million travellers visit malaria-endemic countries annually and about 10,000 cases of malaria are reported after returning home. Due to the fact that malaria is insect vector transmitted, the environment is a key determinant of the spread of infection. Geo-climatic factors (such as temperature, moisture, water quality) determine the presence of *Anopheles* breeding sites, vector densities, adult mosquito survival rate, longevity and vector capacity. Several studies have shown the association between environmental factors and malaria incidence in autochthonous population. The association between the incidence of clinical malaria cases among non-immune travellers and environmental factors is yet to be evaluated. The objective of the present study was to identify, at a country scale (Ivory Coast), the environmental factors that are associated with clinical malaria among non-immune travellers, opening the way for a remote sensing-based counselling for malaria risk prevention among travellers.

**Methods:**

The study sample consisted in 87 cohorts, including 4,531 French soldiers who travelled to Ivory Coast, during approximately four months, between September 2002 and December 2006. Their daily locations were recorded during the entire trip. The association between the incidence of clinical malaria and other factors (including individual, collective and environmental factors evaluated by remote sensing methods) was analysed in a random effect mixed Poisson regression model to take into account the sampling design.

**Results:**

One hundred and forty clinical malaria cases were recorded during 572,363 person-days of survey, corresponding to an incidence density of 7.4 clinical malaria episodes per 1,000 person-months under survey. The risk of clinical malaria was significantly associated with the cumulative time spent in areas with NDVI > 0.35 (RR = 2,42), a mean temperature higher than 27°C (RR = 2,4), a longer period of dryness during the preceding month (RR = 0,275) and the cumulative time spent in urban areas (RR = 0,52).

**Conclusions:**

The present results suggest that remotely-sensed environmental data could be used as good predictors of the risk of clinical malaria among vulnerable individuals travelling through African endemic areas.

## Background

Malaria is an important threat not only for autochthonous populations, but also for non-immune individuals travelling or working in malaria-endemic areas. According to the 2011 international travel and health book, approximately 125 million international travellers visit malaria-endemic countries yearly and over 10,000 cases are reported after returning home [1]. The incidence of imported malaria cases among UK travellers visiting West Africa varied from 52 to 196 cases/1,000 traveller-years between 2003 and 2006 [2]. In a cohort of the French general population, followed from 1994 to 1998, the incidence of malaria imported from endemic areas was 178 cases per 1,000 traveller-years [3]. In the French Armed Forces, the annual incidence rate was 14 per 1,000 person-years in 2006. Amongst French soldiers who served in Ivory Coast between 1998 and 2007, the annual malaria incidence rate ranged from 37 to 388 cases per 1,000 person-years. Preceding works underline the occurrence of several epidemics [4-6] and important contrasts [7] in terms of exposure and incidence between groups travelling in Ivory Coast.

Non-immune travellers should be protected from malaria by chemoprophylaxis and prophylactic measures against mosquito bites (including insecticide-impregnated bed nets, repellents and insecticide-treated long-sleeved clothes and pants). In malaria-endemic areas, the use of most of these prophylactic measures is mandatory for non-immune employees of most major international groups and soldiers.

The effectiveness of these measures is limited by the lack of compliance [8,9] even among military personnel [6,10,11] and even if the chemoprophylaxis is adapted to the chemosusceptibility of *Plasmodium falciparum*[12,13].

The increased number of malaria cases, occurring among traveller populations, has been frequently attributed to behavioural factors. In a previous study [14], the lack of compliance with protective measures was identified as the second most important factor that determined the malaria incidence rate among non-immune travellers, after environmental factors taken into account by the NDVI (*Normalized Difference Vegetation Index*).

Due to the Plasmodium transmission by vectors, the environment is a determinant of malaria. Geo-climatic factors (temperature, moisture, water quality) determine the presence of *Anopheles* breeding sites, the vector densities, the adult mosquito longevity and the vector capacity. Several studies have shown the association between environmental factors and malaria incidence in autochthonous populations [15-17]. Reference methods used for measuring vector transmission levels are entomologic methods but they are not easily implemented. Remotely-sensed indicators have been used as proxy variables to evaluate mosquito densities. Among these indicators used in human health applications, NDVI has been the most commonly used index. It has been associated with the density of vectors and malaria transmission [18-22] and the incidence of clinical malaria cases [22,23]. All these studies have been conducted in autochthonous populations of endemic areas.

Studies concerning non-immune travellers usually neglected environmental factors probably because of difficulties in gathering individual and geographical data for each traveller during his/her trip. Groups travelling to different African countries (where weather and environmental factors are very different) were studied in a previous work [14], which used NDVI as an environmental predictive factor of malaria. The association between the incidence of clinical malaria among non-immune travellers and individual, collective and environmental factors evaluated by remote sensing methods is yet to be evaluated.

The objective of the present study was to identify, at a country scale (Ivory Coast), the remotely sensed environmental factors that were associated with the incidence of clinical malaria among non-immune military travellers (used as a proxy for other types of travellers).

## Methods

### Study population

The itineraries, departure and arrival dates, individual list and dates of birth were obtained from 87 groups of French military personnel on mission to Ivory Coast for short periods of approximately four months at a time, over a period of four years between September 2002 and December 2006.

### Dependent variable

Cases of clinical malaria were recorded by the military weekly disease epidemiological surveillance system and defined as clinical attacks with biological confirmation of plasmodia infection (*i.e.*, positive thin or thick blood smear, positive quantitative buffy coat malaria diagnosis system test, or histidine-rich protein-2 rapid diagnostic tests). The identification of malaria cases (endpoints) was done either before the start date of any new mission to malaria endemic areas, or before January 1^st^, 2007.

### Individual and collective data

Individual (age, manager status) and collective (group accessories, departure and arrival dates) data were provided by the military administration. For each person included, individual variables were created to evaluate his mobility by counting the number of visited sites during the journey and by calculating the average length of his/her stay at each site.

### Environmental data

#### Exposition location

Itineraries were defined by the list of sites where individuals stayed at least one night. Geographical coordinates of the locations visited during the journey were obtained using GPS (Global Positioning System) and were recorded for each group by managers in the military logs. When GPS information was not directly available, the location of the visited places (site, village and town) was attributed using the *National Geospatial-Intelligence Agency* (NGA) and the *US Board on Geographic Names* (USBGN) databases. For this location, two coordinates separated by less than 30 arc seconds (approximately 1 kilometre) were considered as similar.

Each site where a traveller stayed was recorded using (GIS) Arcview^®^ 8.3 (Environmental Systems Research Institute, Redlands, CA), a geographical information system (GIS) software. The World Geodetic System (WGS 84) has been used as reference. Spatial analysis was done with help of Spatial Analyst^®^ module (Environmental Systems Research Institute, Redlands, CA).

#### NDVI

To take into account missing data due to cloud cover [19], NDVI was chosen as the maximal available value over one month. A circle buffer of 1.5 km centred around resting site was extracted in Arcview^®^ GIS and a weighted average of pixels included in the buffer was calculated. This average constitutes the NDVI value of exposure for each person present in a given site for one night. Because previous studies [15,24] suggested association between malaria cases and NDVI measured with a one month or two months lag (*i.e.* before the exposure to infective bites). Value of NDVI up to two months before the stay in each site was also extracted.

To cover the entire study period, two sources were needed for satellite pictures: For the period between September 2002 and February 2004, data concerning Ivory Coast was provided by SPOT 4 satellite launched by CNES (Centre National d'Etudes Spatiales - French space agency). The data was coded in 8 bits and needed to be corrected according to the following formula: [NDVI value = 0.004 x (value of NDVI in the pixel) - 0.1]. Each picture was formatted using the Crop VGT^®^ v1.1 software (University IUAV of Venice, Italy).

For the period between March 2004 and December 2006, data was provided by Terra satellite and his spectoradiometer MODIS IV (*MODerate-resolution Imaging Spectroradiometer*) launched by the NASA (National Aeronautics and Space Administration). The data was coded in 16 bits and needed to be corrected according to the following formula: [NDVI value = value of NDVI in the pixel/10 000]. Pictures were provided in HDF-EOS format (*Hierarchical Data Format - Earth Observing System)* in a sinusoidal projection system. Pictures needed to be rectified with the Modis Reprojection Tool^®^ software (MRT) (Land Processes Distributed Active Archive Centre, Sioux Falls, SD).

#### Weather data

According to previous studies, which described the association between malaria risks and weather parameters in autochthonous populations, and WHO recommendations [15], the following parameters were studied: maximum, mean and minimum temperatures (all in degrees Celsius), cumulated precipitation (in mm^3^ of water), number of consecutive days without rain, evapotranspiration (in mm^3^ of water) and water balance (mm^3^ of water). The data was extracted for each visited site and a map produced using MARS project [25]. The model used by MARS takes into account weather station data and remote sensing information. This technique is described by Beek [26]. For each location (taking into account the amount of time spent in the location), the average value of each of the parameters of interest taken by each pixel in the circle buffer was calculated.

#### Urban mapping

Locations made up of more than 10,000 people or where the population was between 4,000 and 10,000 with more than 50% of household engaged in non-agricultural activities [27] were considered as urban areas in this study. For each traveller, the proportion of days spent in theses urban areas was calculated.

#### Data construction

An independent variable representing the average (computed using the circle buffer surrounding the actual geographical position) of the environmental variables to which each subject was exposed, considering all visited places weighted by the time spent in each place, was built.

For example, for NDVI,

$$\begin{array}{l} {\mathrm{NDVI}}_{\mathrm{mean}}=\frac{\sum_{1}^{i}\left[\mathrm{cumulative}\;\mathrm{NDVI}\;\mathrm{during}\;\mathrm{the}\;\mathrm{journey}\;\right]}{\sum_{1}^{i}\mathrm{days}}\\ \;=\frac{\sum_{1}^{i}\left[\sum_{1}^{j}{\mathrm{NDVI}}_{\mathrm{ij}}\times {\mathrm{NBD}}_{\mathrm{ij}}\right]}{\sum_{1}^{i}\sum_{1}^{j}\mathrm{NB}D_{\mathrm{ij}}} \end{array}$$

with

NDVI_ij_ = NDVI value at location i month j

NBD_ij_ = Number of days spent at location i month j

As suggested by Thomson *et al.*[28], NDVImean was used as a continuous and squared variable. A two-class variable was created using the NDVI threshold of 0.35 associated with increase of malaria risks [14,22,29,30].

For all environmental variables (temperature, rainfall, number of consecutive days without rain), two thresholds were needed (one for the time spent, one for the level of variable). Eleven thresholds were calculated defining the proportion of time spent by each subject in a particular location. Thresholds of time set at 33.3% and 66.6% were identified to maximizing the contrast of the incidence rate in this study. Values of threshold variables were chosen either based on facts from literature (ex: NDVI over 0.35) or by analysis (limit of class maximizing incidence rates).

### Statistical analysis

The incidence rate of malaria was analysed as a dependant variable according to individual and group characteristics using a random effect mixed Poisson regression model, while controlling for the duration of exposure, *i.e.* duration of stay. The model was designed to take into account the intra-group correlations that could exist due to the sampling design by group (group effect was seen as random effect). Resemblance tends to be stronger between subjects within the same group in terms of behaviour and the environment. The Poisson model was also adjusted using a generalized estimating equation (GEE) approach. Random effect and GEE regression models allow the estimation of group specific and population-averaged effects, respectively [31].

First, a descriptive analysis of the independent variables was performed. A bivariate analysis was then conducted by entering each independent variable in a Poisson regression model. Variables were retained for the multivariate analysis when their effect had a p-value less than 0.30 [32]. Because of the numerous environmental variables that were created, a forward stepwise selection procedure was applied. The order in which the variables were introduced depended of their *Akaike Information* Criterion - AIC - obtained in the bivariate analysis; the variable with the lowest score was introduced first. The final model retained significant independent variables (p < 0.05) and their interactions when they were statistically significant and biologically or epidemiologically meaningful. Each variable excluded during the model building process was reintroduced again in the final model to check its contribution and was definitively rejected if it was not significant.

Nested mixed models were compared using the likelihood ratio test and non-nested models were compared using AIC criterion. Group effect was checked using a homogeneity test. The Anscombe residuals [33] were calculated and the statistical quality of the final model was assessed by looking at the adequacy between observed and predicted probabilities of the incidence of clinical malaria. All analyses were performed using STATA 9.0 (StataCorp LP, College Station, TX, USA).

### Ethical clearance

The protocol was approved by the Marseille Ethics Committee (advice no. 02/81, 12/13/2002). All the data was collected anonymously from the epidemiological surveillance system and logs of the military units, so no individual approval needed to be obtained.

## Results

Cohort follow-up corresponded to 572,363 person-days or 18,817 person-months (PM). Among the 4,531 subjects included in the study and distributed in 87 groups, 140 clinical malaria attacks (no severe cases) occurred among 131 persons (incidence rate – IR = 7.4 for 1,000 PM). The mean duration of stay was 126.3 days, ranging from 35 to 149 days (median = 133 d). Mean age at inclusion was 26.1 years, interquartile interval ranging from 22 to 29 years (median = 25 y). Malaria cases occurred between 18 and 522 days after the beginning of the stay (extreme value described for a case of *Plasmodium ovale*) with an average time before the first occurrence evaluated at 140.3 days (CI95% = 124.6 - 156.1). Malaria cases were due to *Plasmodium falciparum* for 108 cases (82%), *P. ovale* for 19 cases (15%) plus four cases of co-infection associating *P. falciparum* with *Plasmodium malariae* for one case (1%) and *P. falciparum* with three cases (2%) due to undetermined species of Plasmodium. Among the 131 individuals with clinical malaria during or after their stay in RCI, 122 and nine persons experienced one and two clinical malaria attacks respectively. Results of the univariate analysis according to clinical status are presented in Table 1.

**Table 1:** individual characteristics according to clinical status (n = 4 531, except other indication)

		**Clinical malaria-free (n =4 400)**	**Clinical malaria (n =131)**	**p**
Age (in years) *		25 [22–29]	25 [22–28]	0.43
Manager †				
	No	3 245 (74%)	109 (84%)	
	Yes	1 141 (26%)	21 (16%)	**0.006**
Year of stay †				
	2003	767 (17%)	8 (6%)	**0.0001**
	2004	1 926 (44%)	86 (66%)	**>0.0001**
	2005	1 288 (29%)	29 (22%)	**0.04**
	2006	419 (10%)	8 (6%)	0.12
Number of sites visited during the stay †				
	<10 sites	3 377 (77%)	92 (70%)	
	≥10 sites	1 023 (23%)	39 (30%)	**0.05**
Average length of stay by site †				
	<15 days	1341(30%)	41 (31%)	
	≥15 days	3 059 (70%)	90 (69%)	0.45
Characteristics of the sites of stay				
Cumulated precipitation (mm of water) *		89 [67–122]	96 [71–157]	**0.008**
Mean Evapotranspiration (mm) *		113 [105–123]	111 [105–128,6]	0.50
Mean Water balance (mm) *		−36 [(−)62 - (+)13]	−25 [(−)52 - (+)59]	**0.008**
Mean Minimum temperature (Celsius) *		20.3 [19.5-22.5]	20.0 [18.9-21.1]	**0.0005**
Mean temperature (Celsius) *		27.5 [25.3-27.5]	27.5 [25.2-27.5]	0.21
Mean Maximum temperature (Celsius) *		32.8 [31.5-35.5]	32,9 [31.4-35.7]	0.95
Mean number of Consecutive dry days § (in days/month) *		3.2 [1.9 – 7.0]	3.1 [1.1 – 6.3]	**0.004**
Proportion of time spent in urban area *		81 [23–100]	64 [15 – 89]	**0.0007**
NDVI mean (0 month of decay) *		0.39 [0.29 – 0.53]	0.39 [0.31 – 0.52]	0.65
NDVI mean (1 month of decay) *		0.38 [0.30 – 0.50]	0.39 [0.30 – 0.52]	0.44
NDVI mean (2 months of decay) *		0.38 [0.31 – 0.49]	0.41 [0.31 – 0.54]	**0.07**

* : Median [interquartile range].

†: Effectif (percent).

$: Number of consecutive days in the month without rainfall.

### Univariate analysis

#### Individual variables

Malaria risks decreased with age (Table 2) from 7.65 malaria access/1,000 PM for people 18–24 years old to 5.19 for those over 40 years. In comparison with the manager category, malaria risks for non-managers was multiplied by 1.19 before 20 years (p = 0.682), by 1.57 between 20 and 24 years (p = 0.074) and by 1.9 over 25 years (p = 0.010). Among managers, age did not modify malaria risks.

**Table 2:** Incidence rate of clinical malaria attacks and estimations from univariate analysis of individual and collective variables (n = 4 531, except other indication)

	**Number of clinical malaria attacks**	**Number of individuals**	**Number of person-days under survey**	**Incidence rate per 1,000 person-month**	**RR**	**95% CI**	**p**
**Age**							
18 - 24 y	66	2 087	262 561	7.65	1		
25 - 29 y	49	1 432	178 907	8,33	1,12	0,77 - 1,64	0,54
30 - 39 y	22	866	110 038	6,08	0,82	0,50 - 1,34	0,43
40 - 56 y	3	137	17 568	5.19	0,74	0,23 - 2,44	0,63
**Manager**							
No	117	3 223	405 122	8.48	1		
Yes	23	1 308	167 240	4.91	0,596	0,380 - 0,935	0,024
**Age and manager status**							
Manager	23	1 162	146 591	4.77	1		
Non Manager							
18 - 19 y	7	256	32 198	6.61	1,19	0,51 - 2,81	0,682
20 - 24 y	53	1 607	203 061	7.94	1,62	0,96 - 2,58	0,074
≥25 y	57	1 491	188 744	9.19	1,90	1,16 - 3,09	0,010
**Year of arrival in Ivory Coast**							
2002, 2003, 2005 or 2006	49	2 519	298 597	4.99	1		
2004	91	2 012	273 766	10.1	1,80	1,10 - 2,97	0,020
**Number of sites visited**							
<10 sites	98	3 469	430 613	6.78	1		
≥10 sites	42	1 062	141 750	9.01	1,454	0,866 - 2,442	0,157
**Average duration of the stay by site**							
<15 days	44	1 382	164 372	8.14	1		
≥15 days	96	3 149	407 991	7.16	0,813	0,486 - 1,360	0,43

CI = confi dence interval; RR = relative risk.

#### Collective variables

Incidence was significantly higher (p = 0,023 with a Relative risk, RR = 1.8; CI95% = 1.16 – 6.76) in 2004 (Table 2) than during the other years (2003 and 2005 to 2006). Mobility variables (number of visited sites, mean duration of stay by site) were not significantly associated with malaria risk (respectively p = 0.16 and p = 0.43).

#### Environmental and meteorological variables

Weather and environmental variables are presented in Tables 3 and 4. Weather variables describing excess of water (rainfall, positive water balance) were significantly associated with an increased risk of malaria. For example, when people spent more than 66% of their stay in locations where water balance was over 15 mm^3^/month (favourable for larva collection), his/her individual risk was multiplied by RR = 1.82 (CI 95% = 1.05 - 3.16; p = 0.032). Conversely, when people spent more than 33% of their stay in urban areas (unfavourable for anopheline breeding sites), the individual risk was divided by 2 (RR = 0.5; CI 95% = 0.30 - 0.86; p = 0.011).

**Table 3:** Incidence rate of clinical malaria attacks and estimations from univariate analysis for a reasoned selection of weather variables (n = 4 531, except other indication)

	**Number of clinical malaria attacks**	**Number of individuals**	**Number of person-days under survey**	**Incidence rate per 1,000 person-month**	**RR**	**95% CI**	**p**
**Cumulated precipitation**							
>180 mm of water by month*							
<33% duration of stay	94	3665	454 512	2,52	1		
≥33% duration of stay	46	866	117 851	4,75	1,69	0,87 - 3,26	0,120
**Water balance**							
> +15 mm of water by month*							
<66% duration of stay	83	3447	425 615 146 748	5,93	1		
≥66% duration of stay	57	1084	146 748	11,81	1,82	1.05 – 3.16	0,032
**Consecutive dry week**							
**number of consecutive dry weeks in month**							
By week (in continuous)					0,93	0,864 - 1,01	0,085
**Evapotranspiration**							
**>150 mm of water by month**							
<33% duration of stay	120	4062	508 236	2,87	1		
≥33% duration of stay	20	469	64 127	3,80	1,31	0,58 - 2,95	0,518
**Maximal temperature**							
**>30**°C							
<66% duration of stay	9	485	64 218	1,71	1		
≥66% duration of stay	131	4 046	508 145	3,14	1,53	0,61 - 3,87	0,368
**Mean temperature**							
**>25**°C							
< 66% duration of stay	55	1 196	158 856	4,22	1		
≥66% duration of stay	85	3 335	413 507	2,50	0,73	0,419 - 1,28	0,271
**Mean temperature**							
By degree Celsius (in continuous)					0,95	0,79 - 1,15	0,629
**Minimal temperature**							
**>20**°C							
< 33% duration of stay	38	693	93 009	4,97	1		
≥33% duration of stay	102	3 838	479 354	2,59	0,56	0,27 - 1,16	0,120
**Urban area**							
<33% duration of stay	57	1 191	157 480	4,41	1		
≥33% duration of stay	83	3 340	414 883	2,44	0,50	0,30 - 0,86	0,011

CI = confi dence interval; RR = relative risk; * estimates calculated with 0 months of decay.

**Table 4:** Incidence rate of clinical malaria attacks and estimations from univariate analysis of remote sensing variables (n = 4 531, except other indication)

	**Number of clinical malaria attacks**	**Number of individuals**	**Number of person-days under survey**	**Incidence rate per 1,000 person-month**	**RR**	**95% CI**	**p**
**NDVI**							
NDVI (M0)* >0,35							
< 66% duration of stay	70	2 391	298 185	7.14	1		
≥66% duration of stay	70	2 140	274 178	7.76	1,10	0,66 - 1,83	0,724
NDVI (M-1)* >0,35							
< 66% duration of stay	77	2 730	342 844	6.83	1		
≥66% duration of stay	63	1 801	229 519	8.35	1,33	0,80 - 2,23	0,268
NDVI (M-2)* >0,35							
< 66% duration of stay	77	2 890	365 385	6.41	1		
≥66% duration of stay	63	1 641	206 978	9.26	1,60	0,97 - 2,66	0,067

CI = confidence interval; NDVI = Normalized Difference Vegetation Index; RR = relative risk; * : M0, M-1 & M-2 correspond to the estimates of NDVI calculated with 0, 1 and 2 months of decay between the remote sensing and the period of stay in the sites.

Concerning NDVI (Table 4), when people spent more than 66% of their time in locations where the mean value of NDVI was over 0.35 (two months before the stay), the risk of clinical malaria increased not significantly by RR = 1.6 (IC95% = 0.97 - 2.66; p = 0.067). Association between NDVI and malaria risk was independent (p = 0.561) of satellite picture source (CNES or NASA). The model fit better when NDVI variable was analysed as a binary variable (*e.g.* time proportion over 66% in areas where NDVI was over 0.35) rather than as a continuous variable (NDVI mean or squared NDVI mean).

### Multivariate analysis

Variables retained in the final mixed multivariate poison regression model are shown in Table 5. There was a significant interaction between age and management status, the risk of clinical malaria became increasingly significant with age only among non-managers. The risk of clinical malaria was significantly associated with the year of the journey (higher risks were observed in 2004 than during the other years), with time spent in sites where NDVI (with a two-month decay) was higher or equal to 0.35 (RR = 2.4), with the number of dry weeks during the previous months of stay in the sites (RR = 0.275) and when the mean temperature was higher than 27°C (RR = 2.4).

**Table 5:** Multivariate analysis based on mixed model Poisson regression (n = 4 531)

	**RR**	**IC95%**	**P**
**Age and manager status**			
Manager	1		
Non Manager			
18 - 19 years old	1,19	0,51 - 2,80	0,685
20 - 24 years old	1,65	1,01 - 2,71	0,046
≥25 years old	1,92	1,18 - 3,12	0,008
**Arrival date in 2004**			
No	1		
Yes	4,99	2,72 - 9,14	< 0,001
**NDVI ≥0,35** (2 month before)			
<66% duration of stay	1		
≥66% duration of stay	2,42	1,48 - 3,97	< 0,001
**Number of consecutive dry week in month, in continuous**	1		
+1 consecutive dry week in month	0,275	0,14 - 0,52	< 0,001
**Mean temperature**			
<27°C	1		
≥27°C	2,40	1,30 - 4,42	0,005
**Group-specific random effect** (i.e. group effect)			0,048

A second model showed that a proportion of time spent in urban areas (environmental variable used in place of NDVI) higher than 33% was significantly associated with a lower risk of clinical malaria (RR = 0.52; IC95% = [0.32 – 0.86] ; p = 0.0011), with little variations in the RR associated to the other covariates included in the model presented in Table 5. Whatever the model, the random effect (i.e. the group effect) was significant (p <0.05). The RR estimates were similar in the random effect model – Poisson and negative binomial regression - and GEE regression models.

## Discussion

Many studies have been conducted in order to identify environmental factors associated with clinical malaria and malaria epidemics in autochthonous and sedentary populations. This study presents the largest cohort of non-immune travellers followed up during trips through heterogeneous endemic areas, that has ever been published (n = 4,531) and has identified environmental factors significantly associated with malaria incidence among non-immune travellers.

Ecological and meteorological parameters play a role not only for the mosquito vector but also for the *Plasmodium* parasite development within anopheline vectors. Among the numerous environmental factors that determine the transmission of malaria, water is one of the most important because it is the basic requirement for the presence of breeding sites hence for the occurrence of the Anopheles vectors.

If water collections appear and persist over time, they can accommodate *Anopheles* mosquito larvae. In this study, direct detection of breeding sites was impossible because they are generally too small or covered by surface vegetation or surrounded by trees. Therefore, surrogate markers for the presence of water were used [34]. Relative air humidity primarily has an impact on the presence and persistence of breeding sites and on the adult mosquito survival time. This parameter can be calculated from meteorological parameters as detailed in Beugnet *et al.*[35], but it should be used with care. Indeed, air humidity is mainly a function of air and temperature, so it can change significantly over the course of the day. The development of vegetation depending on the amount of water available, vegetation indexes could be surrogate markers of precipitation in certain periods and areas [36]. NDVI integrates the combined effects of rainfall, humidity, sunshine, temperature, altitude, land use and land cover [37], all these factors being potentially associated with the presence of sites that are favourable for anopheline vectors, *i.e.;* for the development of larvae (breeding sites) and the survival of adult mosquitoes. NDVI is easily accessible from several captors of satellites. This explains why it has been the most used remotely sensed environmental parameter for forecasting or malaria risk mapping.

Because the larval cycle can range between one and three weeks and sporogonic cycle (*i.e.* from the infection of an adult anopheles during a blood meal on an infected human host up to the invasion of the salivary glands of the vectors by infective sporozoites) can last for a few more weeks, the effect of environmental and meteorological factors on the transmission is delayed. This justified the use of NDVI calculated two months before the period of exposure to malaria transmission in the study sites. As expected, a mean NDVI higher or equal to 0.35 in the sites where the travellers stayed two months after was significantly associated with a higher risk of clinical malaria in this study.

Amongst other variables (other than NDVI) related to the presence of surface water and air moisture (rain fall, dryness, evapotranspiration, hydric balance…), only the number of dry weeks in the month before the start of the visit remained a significant predictor of the incidence of clinical malaria.

Temperature is also one of the main meteorological parameters associated with malaria transmission, and is a determining factor that aids in determining the persistence of breeding sites, the duration of the larval development, the adult mosquito survival rate, the duration of the gonotrophic and sporogonic cycles. Under laboratory conditions, the rate of development of *Anopheles gambiae s.s.* from one immature stage to the next increases at higher temperatures to a peak around 28°C, after which it declines. Adult emergence is optimal between 22°C and 26°C and is inhibited below 18°C or above 34°C [38]. In this study, the risk of clinical malaria increased significantly when temperature mean was higher than 27°C.

Land-cover characteristics drive the spatial and temporal distribution of *Anopheles* species. Thus, dispersal is generally lower (<300 m) in highly-populated urban settings [39,40] than in open rural areas where it can reach several kilometres for some species [41]. Urbanization lowers the contact rate between vectors and hosts (*i.e.* the higher the human density, the lower the risk of mosquito bites at equal mosquito densities), the dispersal of adult mosquitoes, the availability of resting sites in the vegetation for adult mosquitoes, the availability of fresh and unpolluted water collections that could be used as breeding sites [42,43], and the availability of malaria reservoirs, *i.e.* less prevalent infected humans because of the easiest access to effective anti-malarial-drugs. All these reasons could explain why the time spent in urban areas was significantly associated with a lower risk of clinical malaria.

In this study, people with management responsibilities were at lower risk of clinical malaria, probably because of a better awareness about the risks and of their commitment in applying the mandatory protective measures, such as the malaria chemoprophylaxis, the use of insecticide impregnated bed-nets and repellents, and the wearing of long-sleeved clothes at night. The increase of risk of clinical malaria with age among non-managers has been previously shown [14].

Low compliance with malaria preventive measures could be acquired during previous numerous stays in endemic areas by oldest travellers, due to weariness in applying prophylactic measures associated with a false feeling of invulnerability as a result of their escape from clinical attacks during and after their previous exposure to malaria transmission. Moreover, other factors which could not be gathered in this study - retrospective study - could explain the protective status of managers as being due to the difference in activity in the same area of exposition (e.g. managers do not keep guard at night), easy/prioritized access to countermeasures, such as bed nets or insecticides, rest/sleeping area better equipped, frequent contact with medical staff for hierarchical reasons.

A significantly higher risk of clinical malaria was observed in 2004 than during the other years. During the study period, no change in resistance of *P. falciparum* to doxycycline that was used for chemoprophylaxis, or in the malaria control measures could explain the difference. Moreover, no particular changes in meteorological and environmental conditions were noted during this period in RCI. In 2004, the military and field activities were more intense than during the other years. It is possible that fighting operations have hampered the application of the protective measures, such as the use of bed-nets at night, or have led to more frequent forgetfulness of the intake of chemoprophylaxis.

Instructions for applying protective measures remained consistent throughout the study period In RCI. However, differences in compliance with mandatory protective measures or in environmental conditions determining malaria transmission that were not considered in the present study, could explain some differences in the risk of clinical malaria that were observed between groups, resulting in a significant group-effect.

Although this study was performed among French military personnel (i.e. mainly young and healthy population) travelling for a four-month period in tropical Africa, the present results could be directly extrapolated to other groups of non-immune travellers sent to malaria-endemic areas for professional purposes, such as building sites, opencast working, tree-felling, plantations, humanitarian operations within governmental or non-governmental organizations. Extrapolation to individual travellers staying for a shorter duration is less straightforward.

## Conclusion

This study is one of the largest traveller’s cohorts that have ever been constituted for the analysis of individual, collective and environmental factors associated with the risks of malaria. It confirms that several environmental determinants of malaria risks in non-immune travellers were similar to those identified among autochthonous populations. The identification of areas and periods at higher risk of malaria transmission for travellers would allow the implementation of specifically targeted strategies for avoiding the risk (i.e. travelling tour or duration in each site could be modified according to environmental context) or strategies of reinforcement (health information, education and communication) in order to improve compliance with malaria chemoprophylaxis and other prophylactic measures. In case of epidemics among traveller groups the identification of these environmental factors could facilitate the diagnosis of the epidemiological context [44].

## Competing interests

The authors declare that they have no competing interests.

## Authors’ contributions

GT participated in the data collection, the data management and wrote the article. GT, CR, MB performed the statistical analysis. GT, VM participated in the remote sensing data collection, management and spatial analysis. VM, MB participated significantly in the data management. GT, JPB, CR contributed significantly in the preparation of the study and its conception. CR designed the study, took part in the analysis of the data, the discussion and the writing of the article. The final version of the manuscript was seen and approved by all authors.
